# Review of neural rehabilitation programs for dyslexia: how can an allophonic system be changed into a phonemic one?

**DOI:** 10.3389/fpsyg.2015.00190

**Published:** 2015-02-24

**Authors:** Willy Serniclaes, Gregory Collet, Liliane Sprenger-Charolles

**Affiliations:** ^1^Laboratoire Psychologie de la Perception (UMR 8242), Centre National de la Recherche Scientifique and Université Paris Descartes, Paris, France; ^2^Unité de Recherche en Neurosciences Cognitives, Centre de Recherche en Cognition et Neurosciences, Université Libre de Bruxelles, Brussels, Belgium; ^3^Laboratoire de Psychologie Cognitive (UMR 7920), Centre National de la Recherche Scientifique and Aix-Marseille Université, Marseille, France

**Keywords:** dyslexia, determinants of dyslexia, allophonic perception, rehabilitation, perceptual fading

## Abstract

Neural investigations suggest that there are three possible core deficits in dyslexia: phonemic, grapho-phonemic, and graphemic. These investigations also suggest that the phonemic deficit resides in a different mode of speech perception which is based on allophonic (subphonemic) units rather than phonemic units. Here we review the results of remediation methods that tap into each of these core deficits, and examine how the methods that tap into the phonemic deficit might contribute to the remediation of allophonic perception. Remediation of grapho-phonemic deficiencies with a new computerized phonics training program (GraphoGame) might be able to surpass the limits of classical phonics training programs, particularly with regard to reading fluency. Remediation of visuo-graphemic deficiencies through exposure to enhanced letter spacing is also promising, although children with dyslexia continued to read more slowly than typical readers after this type of training. Remediation of phonemic deficiencies in dyslexia with programs based solely on phonemic awareness has a limited impact on reading. This might be due to the persistence of a covert deficit in phonemic perception. Methods based on slowed speech enhance the perception not only of phonemic features but also of allophonic features, and this is probably why they have not been found to be effective in meta-analyses. Training of phonemic perception with a perceptual fading paradigm, a method that improves precision in identification and discrimination around phonemic boundaries, has yielded promising results. However, studies with children at risk for dyslexia and dyslexic adults have found that even when behavioral data do not reflect allophonic perception, it can nevertheless be present in neural recordings. Further investigations should seek to confirm that the perceptual fading paradigm is beneficial for reading, and that it renders perception truly phonemic.

## THE THREE SOURCES OF DYSLEXIA

Developmental dyslexia is a specific learning disability characterized by difficulties in the acquisition of low-level reading skills: i.e., accurate and/or fluent word recognition and decoding skills (Lyon et al., 2003). Developmental dyslexia affects about 5–10% of the population (Shaywitz et al., 1990; Peterson and Pennington, 2012). Low-level reading skills, especially decoding skills, are chiefly a matter of relating the basic units of the written language (letters and groups of letters called graphemes) to the basic units of the spoken language (phonemes). Children with dyslexia experience great difficulties in learning grapheme–phoneme associations and, once acquired, these associations remain suboptimal (Lachmann and van Leeuwen, 2014). Dyslexic children do not read fluently and expend much more energy in reading than typical children (Shaywitz and Shaywitz, 2005; Sprenger-Charolles et al., 2006; Blomert and Vaessen, 2009).

The most obvious possible reason for dyslexics’ problem in establishing grapheme–phoneme relationships is a deficiency in cross-modal neural mechanisms (Blomert, 2011). Evidence of areas responsive to the simultaneous presentation of letters and speech sounds in the temporal cortex (superior temporal sulcus, STS and superior temporal gyrus, STG) has been presented. Furthermore, it has been shown that when a letter and sound occur within the same narrow time-window, letters influence the processing of speech sounds (van Atteveldt et al., 2004). This and other related findings (Blomert and Froyen, 2010) suggest that letter–sound integration is performed by specialized neural processes. Such cross-modal integration also occurs in dyslexic children with 4 years of reading instruction, but the influence of print on sound perception is much weaker for them than for age-matched controls, and it only appears when the letter is presented much earlier than the sound (Froyen et al., 2011).

A specific failure in the simultaneous binding of letters with speech sounds is not the only possible cause of dyslexia, however. There are two other main reasons why grapheme–phoneme associations might be deficient in the absence of specific binding problems. The first factor that might also affect grapheme–phoneme associations is a deficiency in the visual processing of letters^1^. This hypothesis has been formulated in various different ways, and might be explained in the framework of a recent theory (the “neuronal recycling hypothesis”: Cohen and Dehaene, 2004; Dehaene, 2014). The theory attributes fluent word recognition to a specific brain area of the left hemisphere [dubbed the “visual word form area (VWFA)”], which was initially devoted to visual processing requiring a level of acuity similar to that needed by letter processing but which, in recent human history, has been recycled for letter perception. However, a recent survey indicates that, besides being used for visual word perception, the VWFA has maintained its original function in processing other visual stimuli (Vogel et al., 2014; see also Dehaene, 2014). Vogel et al. (2014) also noted evidence that activity in the occipito-temporal cortex is strongly correlated with the dorsal attentional network. This is in accordance with several studies that point to the role of visuo-attentional deficits in part of the dyslexic population (e.g., Franceschini et al., 2012; Lobier et al., 2012).

A second factor that might also affect grapheme–phoneme associations is a deficiency in the phonological processing of speech sounds. Children with dyslexia often exhibit a lack of phonemic awareness: i.e., a problem with the ability to segment words into phonemes, a skill which is required to learn to read in an alphabetic system, but not required to learn to speak (Liberman et al., 1974). A deficit in phonemic awareness might be responsible for difficulties in relating these units to graphemes (for a review, Melby-Lervåg et al., 2012). However, the deficit in phonemic awareness is probably the consequence of a more drastic difference in the mode of speech perception. Perceiving speech sounds in terms of subphonemic units (allophones) induces serious problems for relating them to phoneme-sized graphical units. This is the possibility raised by the “allophonic” theory of dyslexia (Serniclaes et al., 2004).

**FIGURE 1:**
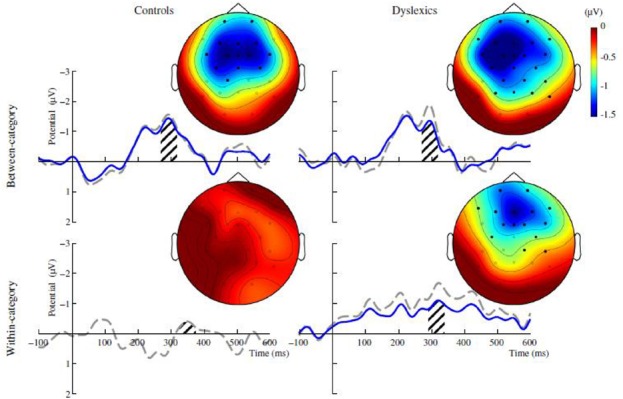
**Mismatch negativity (MMN; a pre-attentive neurophysiological response, a component of the event-related potential) for a phonemic contrast (above) and for an allophonic contrast (below) in adult dyslexics (right) and controls (left).** Adapted from Noordenbos et al. (2013, Figure 3).

There is growing evidence that individuals with dyslexia discriminate between allophonic variants of the same phoneme, whereas typical-reading controls do not perceive such distinctions (Bogliotti et al., 2008; Noordenbos et al., 2012a,b, 2013, for a comprehensive review of the available evidence; Serniclaes and Sprenger-Charolles, 2015). Even when there is apparently no behavioral manifestation of allophonic discrimination (e.g., Messaoud-Galusi et al., 2011), it can nevertheless be present in the brain. This has been evidenced by the results of studies conducted in Dutch with either children at risk for dyslexia or adults with dyslexia (Noordenbos et al., 2012b, 2013; see Figure 1). The lack of a behavioral manifestation of allophonic processing, that in fact takes place at the neural level, suggests the involvement of inhibitory processes. Such processes would inhibit the neural responses to allophonic contrasts so that only the neural responses to phonemic contrasts would be available for emitting the behavioral responses. According to a PET study with French adults with dyslexia, such processes might take place in the frontal cortex in the inferior frontal gyrus (IFG) close to Broca’s area (Dufor et al., 2009). Inhibition is costly in terms of metabolic resources that then are not available for reading, a possible cause for the slow and laborious performance in word recognition and decoding that characterize dyslexia (Shaywitz and Shaywitz, 2005; Sprenger-Charolles et al., 2011). Reduced metabolic resources on reading might for instance slower the transmission of the phoneme percept from the frontal cortex to the areas of the temporo-parietal cortex that are responsible for grapheme–phoneme associations.

**FIGURE 2:**
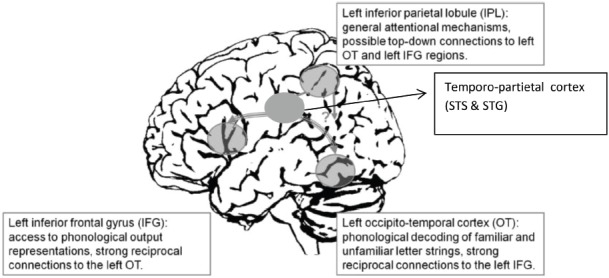
**Left-hemisphere reading network (adapted from Richlan, 2012, Figure 1).** The graphemic representations (occipito-temporal cortex) are related to phonemic representations (frontal cortex: IFG) via grapheme–phoneme bindings (temporo-parietal cortex). Attentional mechanisms (IPL) might act upon both phonemic and graphemic representations.

In summary, the processes involved in low-level reading skills are carried out by a neural network (Figure 2) that relates graphemic representations (occipito-temporal cortex) to phonemic representations (frontal cortex) via grapheme–phoneme bindings (temporo-parietal cortex). In turn, the three sources of dyslexia could be summarized as follows: a grapho-phonemic deficit due to a lack of strong and timely grapheme–phoneme associations, a graphemic deficit due to a failure to combine letters (or graphemes) into word representations, and an audio-phonemic deficit arising from an allophonic mode of speech perception.

Each of these three possible core deficits has prompted attempts at remediation. Here we first review the results of the available remediation methods. We then see how the remediation of allophonic perception might contribute to overcome some of the limitations of these methods.

## REMEDIATION OF GRAPHEME–PHONEME ASSOCIATIONS

As a failure to associate graphemes with phonemes is the most proximal cause of dyslexia, intervention studies should primarily aim to enable or improve the learning of grapho-phonemic associations. Not surprisingly, then, different kinds of grapho-phonemic training have been used in attempts to aid in reading acquisition and remediate dyslexia.

The results of a first meta-analysis (Ehri et al., 2001a, see also the results of the long-term longitudinal study of Johnson et al., 2012) indicate that systematic phonics instruction (mainly when based on grapheme–phoneme correspondences and not on rhyme units for instance) can improve the acquisition of low- and high-level reading skills, especially when training begins early and in children at risk for reading disability; the benefits of such training are lesser in children with reading disabilities (dyslexics). However, the results of two recent meta-analyses of training studies (McArthur et al., 2012; Galuschka et al., 2014) indicate that classical phonics instruction is the only treatment approach whose efficacy in children and adolescents with reading disabilities is statistically confirmed. Furthermore, as noted by Gabrieli (2009), only about 50% of dyslexics retain the reading progress they make after explicit and systematic instruction in decoding strategies and phonemic awareness, and those who do retain their gains do not attain the fluent reading competency of typical-reading children.

A new computerized phonics training program (Grapho-Game) appears to be able to surpass the limits of classical phonics training programs, especially with regard to reading fluency. The aim of GraphoGame is to strengthen the binding between the orthographic and phonological encodings of words (for a review, Richardson and Lyytinen, 2014). The GraphoGame method is mainly based on the training of grapheme–phoneme correspondences. This method progresses from the simultaneous and repeated presentation of grapheme–phoneme correspondences (first in isolation, then included in syllables, and afterward in words) to fluency training with words and sentences. GraphoGame’s effectiveness in improving reading acquisition has been demonstrated in several studies conducted in languages with various levels of orthographic transparency: Finnish (Saine et al., 2010, 2011), German (Brem et al., 2010), and English (Kyle et al., 2013).

In the Finnish study, after a screening of 166 first graders [with tests assessing letter knowledge, phonological awareness, and rapid automatic naming (RAN) of letters, digits, or pictures of frequent words], the lowest-achieving 30% were randomly assigned to two different remedial interventions (25 children in each group): a regular remedial phonics intervention (RRI; Note^2^) or a computerized assisted intervention using GraphoGame (computer assisted regular remedial intervention, CARRI), both with four weekly sessions of 45 min for 28 weeks. These two groups were compared to the remaining children, who received “mainstream” reading instruction. For word reading fluency, at the end of the first grade, the CARRI group outperformed the RRI group, while both differed from the mainstreamers. One year later the difference between the CARRI group and the RRI group was still significant, but not the one between the CARRI group and the mainstreamers. Moreover, at that time, only three children from the CARRI group (11%) still presented a severe deficit in word reading fluency, versus 11 children from the RRI group (44%). Although these data are drawn from on a small number of participants, they are of interest, especially those from the RRI group, which are very similar to those reported by Gabrieli (2009) about the percentage of dyslexics who are “resistant” to classical phonological interventions. In addition, the CARRI group’s gradual gains in word reading fluency indicate that children at risk for reading disability can reach the level of mainstream students. However, they require much more time to reach that level. These Finnish results were replicated in a language with a non-transparent orthography (English, Kyle et al., 2013). However, the interpretation of the results of that study is limited by the fact that no individual data were provided and no fluency evaluations were performed.

**FIGURE 3:**
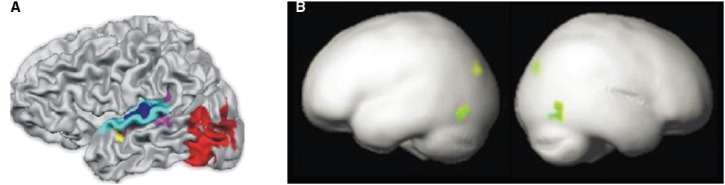
**(A)** Letter–sound association area (in blue) and VWFA (in red) in the left STS-STG (adapted from Blomert, 2011, Figure 1). **(B)** GraphoGame training effects (in green; adapted from Brem et al., 2010, Figure 2).

In another training study (Brem et al., 2010), functional magnetic resonance imaging (fMRI) data were collected from 16 German-speaking kindergarteners from Switzerland trained with both GraphoGame and a non-linguistic number-knowledge control game (duration of each of the two training programs: less than 4 h per week over 8 weeks). The results showed that behavioral improvements were accompanied by activity changes in the VWFA in the left occipito-temporal cortex (Figure 3). This contrasts with (later) findings by Blomert (2011) showing that the neural site of letter–speech sound bindings is located in the left temporal cortex suggesting that the results of a grapho-phonemic training method such as GraphoGame should primarily affect the letter–sound area. An effect of grapho-phonemic training on the VWFA is not surprising because the development of that areas depends on reading instruction (Dehaene et al., 2010). However, it nevertheless seems that GraphoGame should primarily impact the neural site of letter–speech sound bindings. One possible explanation to this discrepancy is that in Brem’s study the effects of GraphoGame were assessed by comparing sensitivity to letters vs. other visual symbols. Possible changes in the sensitivity to letter–sound bindings, that should take place in temporo-parietal cortex, were thus not directly evaluated.

Instead of directly arising from a deficit with complex written symbols, the deficit in visual-auditory integration might arise from remote lower-level deficits. Training 7-year-old dyslexic children to associate elementary sound features (e.g., duration) with simple visual features (e.g., length) has been found to have positive effects on reading skills (Kujala et al., 2001). A recent study evidenced impaired audio-visual integration of low-level stimuli in dyslexic adults (Harrar et al., 2014). However, another recent study with adolescent dyslexics found that they exhibited specific problems with grapho-phonemic conversions even though their basic audio-visual integration mechanisms seemed to be intact (Kronschnabel et al., 2014). Whatever conclusions are ultimately drawn on this point, remediation methods might benefit from a better understanding of the processes involved in grapheme–phoneme integration.

## REMEDIATION OF VISUO-GRAPHEMIC DEFICIENCIES

There have been several attempts to remediate graphemic deficits in dyslexia through the facilitation of low-level visual processing. A study with Italian and French children with dyslexia (Zorzi et al., 2012) showed that simple exposure to enhanced letter spacing led to improved reading accuracy and speed in both linguistic groups (34 to 40 children with dyslexia per language group, about 10 years old, 2-month follow-up). However, a subsequent study with Spanish children (Perea et al., 2012) found that the reading speed of children with dyslexia after exposure to enhanced letter spacing remained lower than that of typical readers (18 children with dyslexia, about 12 years old). This limitation might be due to the fact that the existing visuo-graphemic interventions do not tap into the perception of letters as visual categories. Letter enhancement taps into low-level visual processing, so that it magnifies not only the distinctions between letters that are relevant for word decoding, but also a host of graphical details that do not contribute to letter recognition.

Other remediation studies have aimed at improving visuo-attentional performance using video games (e.g., Green and Bavelier, 2012). Franceschini et al. (2013), used “action” and “non-action” video games, differing in cognitive load and speed requirements, and compared their effects on reading. Two groups of 10 Italian children with dyslexia, of about 10 years of age, were randomly assigned to the “action” and “non-action” training groups (12 h at 80 min per day). The results showed significant improvement in reading performance only with the “action” training. When measured with a speed/accuracy score, the resulting progress in reading was equivalent to one year of spontaneous reading development. However, the study participants did not seem to present phonological deficits, meaning that the benefits of visuo-attentional training cannot be generalized to the whole dyslexic population. More importantly, studies in this area are still too rare to allow generalizations.

## REMEDIATION OF PHONOLOGICAL DEFICIENCIES

### INTERVENTIONS AIMED AT IMPROVING PHONEMIC AWARENESS

Meta-analyses indicate that early phonemic awareness training helps children at risk for reading disability to acquire word-level reading skills, but such training has lesser effects in those who have already developed reading difficulties (Ehri et al., 2001b; National Institute for Literacy, 2008). These meta-analyses also highlight the fact that such training is very effective only when the letters (or graphemes) are presented together with the corresponding phonemes: remediation methods that train phonemic awareness alone have a limited impact on reading, and especially fluent reading. Furthermore, interventions using both grapheme–phoneme training and phonemic awareness training have neural effects in the left hemisphere reading network (Démonet et al., 2004), including the VWFA (Brem et al., 2010).

### INTERVENTIONS AIMED AT IMPROVING LOW-LEVEL AUDITORY PROCESSES

Numerous auditory training methods have been proposed (for a review, Collet et al., 2014). For instance, Earobics^®^ (Morrison, 1998; Diehl, 1999) is a computer-assisted training program which aims to improve reading skills by improving children’s sound perception, memory, and phonological awareness. This program consists of a number of tasks, such as phoneme identification and discrimination and rhyme judgments. It has been widely used in the teaching of reading in American schools, but also in children with language learning difficulties specifically. Using this program with dyslexic children, Russo et al. (2005) showed a significant improvement of neural synchrony in the auditory brainstem in children who had received the training, while those who had not received this training showed no such changes. These results suggest that dyslexic children derive some benefits from this training, and that these benefits are also seen at the level of subcortical structures.

In the same vein, other studies have attempted to develop procedures to improve the auditory-perceptual abilities of children with learning disabilities. Merzenich et al. (1996) and Tallal et al. (1996), hypothesized that dyslexic children have a temporal processing disorder that could be remediated through auditory training, developed a computerized training program known as Fast ForWord^®^ (Scientific Learning Corporation, Oakland, CA, USA). The program consisted in a succession of tasks such as the comparison or identification of sounds, phonemes, syllables, and words with variations in acoustic parameters such as duration and frequency, and was recommended during a period of 6 to 8 weeks (100 min a day, 5 days a week). Tallal et al. (1996) found that such auditory training had positive effects on the perception and understanding of speech. However, meta-analytic reviews indicate that these remediation attempts do not have reliable effects on reading performance (e.g., Strong et al., 2011).

### INTERVENTIONS AIMED AT REMEDIATING ALLOPHONIC PERCEPTION

Contrary to typical phonemic perception, which combines different auditory features and weights them differently as a function of contextual features, allophonic perception uses these features independently and irrespective of context. For instance, French dyslexic children are sensitive to two different features that are perceived independently by the pre-linguistic child but are dynamically combined for separating voiced and voiceless consonants in French, with weights depending on the syllabic context (Bogliotti et al., 2008). Remediation of allophonic perception is intrinsically difficult, because it means modifying processes that allow the child to perceive speech sounds, albeit in a non-optimal way. There is no need to tap into auditory processes to remediate allophonic perception, because the use of allophonic units is not a matter of auditory feature perception as such but a matter of combining auditory features in way that is relevant for speech perception.

Discriminant training of minimal pairs (Hurford, 1990; Hurford and Sanders, 1990; Veuillet et al., 2007) might be of some help, but it has no straightforward implications for phonemic perception. Discriminating two different phonemes is supposed to be achieved with a phonemic boundary, but it can also be achieved through one of the several allophonic boundaries that separate these two phonemes. Similarly, the deletion of the initial phoneme from a word and other “phonemic awareness” performances are normally achieved with a phonemic cut-off point, but they can also be achieved with allophonic cut-off points. What is needed to remediate allophonic perception is to modify the boundaries that are used to discriminate and segment speech sounds, something that is not guaranteed with classical methods.

Until now, only a handful of studies have tried to remediate allophonic perception in people affected by dyslexia. Bogliotti (2005) trained severely impaired dyslexic children (five trained children and five untrained controls between 8 and 10 years of age) to identify allophonic variants of the same phoneme with the same label (following a procedure initiated by Guenther et al., 1999). The training improved the accuracy of phoneme identification, but it did not improve discrimination around the phoneme boundary. On the contrary, the training gave rise to discrimination peaks around allophonic boundaries. Allophonic discrimination was probably present in these children before training, but it only became apparent in behavioral responses after training. This suggests that allophonic perception is indeed highly resistant to training.

Recently, Collet et al. (2012) developed a new method, adapted from the “perceptual fading” training program (Jamieson and Morosan, 1986). The basic approach was to progressively reduce the acoustic distance between two stimuli as a function of each child’s individual performance. This method aimed to teach children to discriminate fine acoustic differences between two different phonemes. During the study, the stimuli varied along a də/tə voice onset time (VOT) continuum, and the acoustic difference in VOT around the French VOT boundary was progressively reduced. At each stage, these pairs of different phonemes were mixed with other random pairs composed of identical phonemes. The task required the child to determine whether the pairs sounded alike or different. After each answer, the child received positive or negative visual feedback (green or red screen) on accuracy. As soon as the child’s performance was stabilized above 75%, the acoustic distance between phonemes was reduced in the next training step. This transition thus occurred when minimally distinct phonemes in acoustic terms were discriminated above chance level.

**FIGURE 4:**
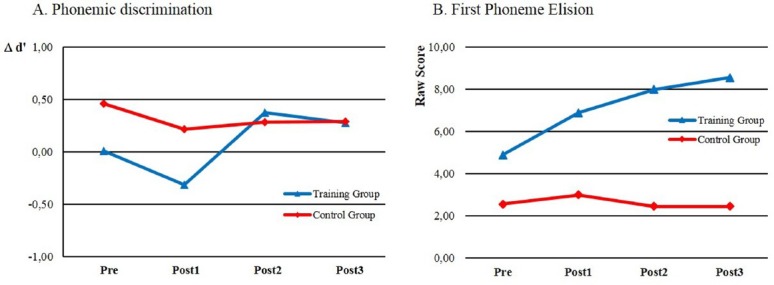
**Effects of phonemic discrimination training on SLI children with impaired reading skills: (1) on the difference in size (scaled in *d*′; Macmillan and Creelman, 2005) between phonemic and allophonic discrimination peaks (A; adapted from Collet et al., 2012, Figures 3 and 4); and (2) on first phoneme elision performance, scored from 0 to 10 (B; adapted from Collet et al., 2012, Figure 6).** Data were collected during four different sessions: for the training group, at the beginning of training (pre), in the middle of training (Post1), at the end of training (Post2), and 1 month post-training (Post3), and according to the same timeline for the control groups.

The total duration of the training was about 18 h (2 × 9 sessions of about 25 min each). Eighteen 9-year-old children with specific language impairment (SLI; which delays the mastery of oral language skills) who also had impaired reading and spelling skills (at least 1.5 standard deviations below the mean for their age) participated in the study. These children were randomly assigned to either a training group or a control group of equal size. Results showed that perceptual fading improved both discrimination and identification performance in these children. Allophonic discrimination peaks emerged after the initial training sessions, just as in the previous study with dyslexic children (Bogliotti, 2005, see above), but they were progressively replaced by phonemic peaks in the later sessions (Figure 4). Importantly, phonemic awareness considerably improved after perceptual training. Unfortunately, reading performance was not evaluated at the end of the training.

Several important questions remain open concerning the impact of phonological remediation with perceptual fading (hereafter audio-phonological remediation, APR). One question is whether APR contributes to remediating reading deficits in dyslexia. The fact that the SLI children studied by Collet et al. (2012) also began with a reading deficit suggests that APR will also improve phonemic awareness in dyslexic children. And although there is presently no (published) evidence in support of the benefits of APR for reading, given the strong effects of APR on phonemic awareness it should also have at least some impact on reading performance. Preliminary results from APR training with dyslexic children suggest that this type of training is indeed beneficial for reading and spelling performance (work in progress).

Another question is whether APR truly transforms an allophonic system into a phonemic one. Recall that discrimination of allophonic peaks can be completely absent from behavioral responses even when it is present in neural processing (in children: Noordenbos et al., 2012b; in adults: Noordenbos et al., 2013). APR might thus give rise to a hybrid system that appears to be phonemic but that remains basically allophonic. Still another question is whether APR is beneficial for individuals with dyslexia who do not exhibit allophonic perception at the behavioral level although their neural processing is allophonic. Studies examining neural activity are needed to clarify these points.

## CONCLUSION

Among the various methods that have been used in attempts to remediate dyslexia, those involving grapho-phonemic training are currently the most successful. However, as there are three possible sources of dyslexia (phonological, grapho-phonemic, and graphemic) several different methods need to be tried. Graphemic methods are successful in part of the dyslexic population. Phonological remediation based on phoneme awareness alone has only a limited effect on reading, especially in dyslexic children. A possible reason for these limitations is that training a child to manipulate phoneme-like segments does not guarantee a change in the way the child perceives these segments. Some recent studies suggest that a subset of people with dyslexia perceive speech in allophonic segments instead of phonemic ones, a distinction that is not captured by phoneme awareness tasks. A new method of phonological remediation that is specifically designed to change an allophonic mode of speech perception into a phonemic one is promising, although its effects on reading need to be confirmed.

### Conflict of Interest Statement

The authors declare that the research was conducted in the absence of any commercial or financial relationships that could be construed as a potential conflict of interest.

## Footnotes

1 One of the possible indicators of a visual source of dyslexia is the absence of problems in non-word reading, which is fairly rare in the dyslexic population (see Sprenger-Charolles et al., 2011, for a review of the literature, with a meta-analysis of 300 cases of dyslexics). However, the phonological deficit might be a secondary consequence of a more basic visual deficit, and there are also positive indicators of visual problems, such as those in parallel letter-string processing (Lobier et al., 2012).

2 The RRI included activities linking reading, spelling and phonology, training of word segmentation, training of decoding and spelling, and vocabulary training.
